# Cost-Effective Biochar Produced from Agricultural Residues and Its Application for Preparation of High Performance Form-Stable Phase Change Material via Simple Method

**DOI:** 10.3390/ijms19103055

**Published:** 2018-10-07

**Authors:** Yan Chen, Zhixing Cui, Han Ding, Yechao Wan, Zhibo Tang, Junkai Gao

**Affiliations:** School of Port and Transportation Engineering, Zhejiang Ocean University, Zhoushan 316022, China; chenyan@zjou.edu.cn (Y.C.); 15195951772@163.com (Z.C.); dinghan940519@163.com (H.D.); 18368090669@163.com (Y.W.)

**Keywords:** phase change material, biochar, polyethylene glycol, agricultural residues

## Abstract

A new form-stable composite phase change material (PEG/ASB) composed of almond shell biochar (ASB) and polyethylene glycol (PEG) was produced via a simple and easy vacuum impregnation method. The supporting material ASB, which was cost effective, environmentally friendly, renewable and rich in appropriate pore structures, was produced from agricultural residues of almond shells by a simple pyrolysis method, and it was firstly used as the matrix of PEG. Different analysis techniques were applied to investigate the characteristics of PEG/ASB, including structural and thermal properties, and the interaction mechanism between ASB and PEG was studied. The thermogravimetric analysis (TGA) and thermal cycle tests demonstrated that PEG/ASB possessed favorable thermal stability. The differential scanning calorimetry (DSC) curves demonstrated that the capacities for latent heat storage of PEG/ASB were enhanced with increasing PEG weight percentage. Additionally, PEG/ASB had an excellent thermal conductivity of 0.402 W/mK, which was approximately 1.6 times higher than that of the pure PEG due to the addition of ASB. All the study results indicated that PEG/ASB had favorable phase change properties, which could be used for thermal energy storage.

## 1. Introduction

Energy demand and consumption are increasing noticeably and continuously due to the rapid development of society and the economy. Therefore, the methods used to promote energy utilization and conservation technologies have attracted great attention in various industries. Recent years have seen an increasingly intense research effort devoted to the study and application of phase change materials (PCMs), which show favorable abilities to store and release abundant thermal energy during the course of phase change [1,2,3]. PCMs are important materials in the field of latent heat storage due to their high stored energy density and chemical stability [4,5]. Currently, PCMs can be divided into three main categories, including organic PCMs, inorganic PCMs and their mixtures [6,7,8]. Organic PCMs show the advantages of a reasonable price and good phase change behaviors, such as appropriate phase change temperatures and enthalpies [5,9]. As a typical organic PCM, polyethylene glycol (PEG) has been confirmed to be a promising material and has received wide attention due to characteristics such as excellent latent heat capacity, suitable melting temperature, and prominent chemical stability [9,10]. However, low heat conductivity as well as the problem of liquid leakage during phase transitions have become the two major shortcomings of PEG that limit its applications to a certain extent [11,12].

An effective method to address these problems is to create form-stable composite phase change materials (CPCMs) that encapsulate PCMs by porous supporting materials, including graphene [13], diatomite [14], activated carbon (AC) [15,16,17], expanded graphite [18], silica [19,20,21] and some polymers. Among these materials, AC has been favored by researchers for use as the matrix of PCMs because of it features a porous structure and low density [15,17]. Among the studies of AC in form-stable CPCMs, Feng et al. [16] prepared PEG/AC CPCMs through the blending and impregnation methods. They used several techniques to research the thermal behaviors of CPCMs and found that the PEG content in the composites as well as the molecular weights strongly affected the structural and thermal characteristics of CPCMs. Chen et al. [17] used AC as supporting material to adsorb liquid lauric acid (LA) for the preparation of form-stable CPCMs. They found that the thermal stability and thermal conductivity of LA/AC CPCMs were improved by the addition of AC compared to those of the pure LA, and they concluded that LA/AC CPCMs could be applied in different aspects of energy storage, including solar energy, heat recovery programs and building energy conservations. However, activated carbon still suffered from the drawbacks of high production cost, nonrenewable manufacture sources and regeneration difficulty [22].

Biochar, as a type of carbon-rich material, can be produced via pyrolysis treatment of biomass like agricultural or forests residues under an oxygen-limited environment [23,24,25]. Currently, biochar has become a research hotspot because of the merits, such as its relative low production cost, highly functionalized surface and porous structure [26,27,28,29]. To date, various kinds of biochar have been produced from different raw materials, and many biochars were applied in the field of heavy metal ions removal [27,28,30]. Dawood et al. [22] prepared biochar from pine cones and found that it was an effective adsorbent as measured by the removal of methylene blue dye and Ni(II) ions in aqueous solutions. Jia et al. [31] selected rice husk and cotton straw to prepare biochars for the adsorption of Cd(II). They found that both two biochars owned rich functional groups on their surface, and the adsorption ability of biochars for Cd(II) could be improved with the increase of pyrolysis temperature. They concluded that the large surface area of biochar was significant for Cd(II) adsorption. Li et al. [32] used rape straw to obtain biochar through different modification processes and found that the biochar adsorption ability of Cd(II) was heightened after KMnO_4_ impregnation. However, research reports about form-stable CPCMs based on biochar are still rare, and the use of biochar as the foundation of form-stable CPCMs production could not only solve the liquid leakage problem and improve the thermal conductivity of organic PCMs but could also make full use of agricultural solid wastes, reducing the preparation cost of form-stable CPCMs. Therefore, further studies of organic PCMs immobilizing in biochar are currently still required.

Thus, in this work, the environment friendly, renewable and cost-effective biochar (ASB) with appropriate pore structures was prepared with agricultural residues of almond shells by a simple pyrolysis method, and then it was used as the matrix of PEG to develop a new form-stable composite phase change material (PEG/ASB) through a simple vacuum impregnation method. Different techniques were used to investigate the characteristics of PEG/ASB CPCMs such as structural properties and thermal properties. The study results suggested that PEG/ASB had favorable phase change properties, and that it could be used for thermal energy storage.

## 2. Results and Discussion

### 2.1. Structural Properties

#### 2.1.1. Microstructure of ASB

**Figure 1:**
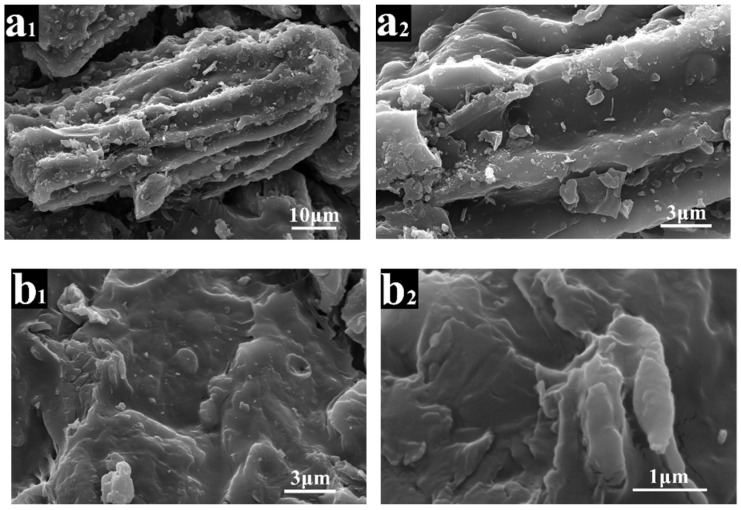
Scanning electronic microscopy (SEM) images of almond shell biochar (ASB) (**a_1_**,**a_2_**) and PEG/ASB CPCM with 60% theoretical PEG content (CPCM3) (**b_1_**,**b_2_**).

The scanning electronic microscopy (SEM) images of ASB and PEG/ASB CPCM with 60% theoretical PEG content (CPCM3) are shown in Figure 1. As shown in Figure 1(a_1_,a_2_), the almond shell biochar contained many particles of carbon with an abundance of pores. This structure suggested that ASB may have a considerable porosity and surface area, which may be beneficial for the adsorption and storage of PEG. Examination of Figure 1(b_1_,b_2_) shows that many of the ASB pores were filled, which meant that ASB adsorbed and fixed PEG successfully.

**Figure 2:**
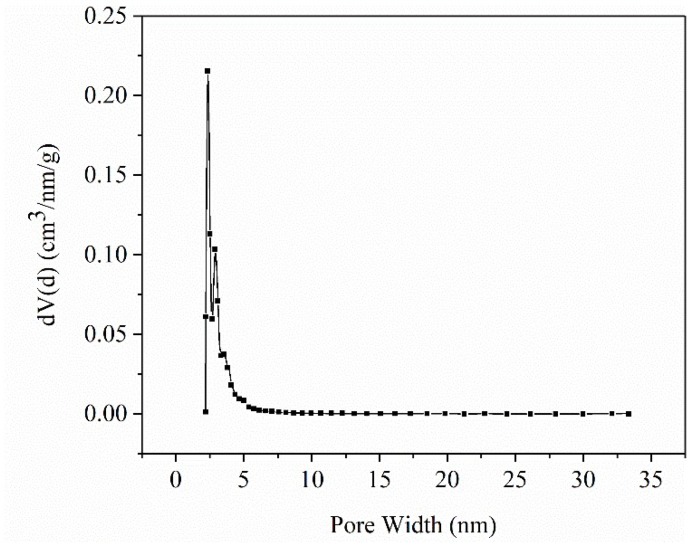
Pore size distribution curve of ASB. dV(d): Pore size per nanometer spacing, pore volume per gram of biochar.

The pore size distribution curve for ASB is displayed in Figure 2. The surface area of ASB was 291.21 m^2^/g, and the total pore volume was 0.17 cm^3^/g, suggesting that ASB may own favorable adsorption ability. It can be clearly seen from Figure 2 that there were two obvious high peaks at the pore widths of 2.35 nm and 2.89 nm, which correspond to most pore diameters, and the final actual average pore diameter was 2.33 nm. The outcome of the pore size distribution showed that ASB was a typical mesoporous solid material [33]. In conclusion, biochar in this study showed favorable pore structure properties that are the same as those of the many other carbon materials and may be used as a reliable adsorbent material for the adsorption and storage of PEG. Moreover, biochar pyrolysis is influenced by some factors, such as the heating rate, pyrolysis temperature and residence time at the pyrolysis temperature [34,35,36]. Therefore, according to the pore characteristics of ASB, the pyrolysis parameters used in this work were considered appropriate.

#### 2.1.2. Leakage Test of PEG/ASB Composites

**Figure 3:**
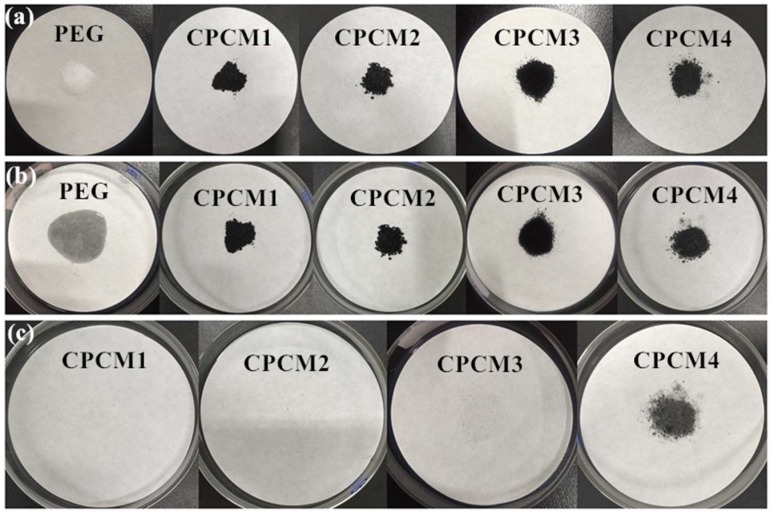
Leakage test of polyethylene glycol (PEG) and composite phase change materials composed PEG and ASB (PEG/ASB CPCMs); (**a**) pictures of samples at indoor temperature; (**b**) pictures of samples after leakage tests; (**c**) pictures of leakage trace after the removal of samples.

The leakage test results of PEG/ASB CPCMs are displayed in Figure 3. Figure 3a shows the normal solid state of samples at indoor temperature. It can be clearly seen from Figure 3b that pure PEG completely melted and could not retain a stable form at 65 °C. On the contrary, as shown in Figure 3b, PEG/ASB CPCMs with different theoretical PEG contents including 40% (CPCM1), 50% (CPCM2) and 60% (CPCM3) all remained in an original stable solid state after leakage tests, and it can be found in Figure 3c that there was no liquid PEG leakage trace on the filter papers of all the three samples. The reason of this phenomenon was that the porous structure of ASB could bring capillary and surface tension forces for PEG molecules, and then PEG was protected by ASB and could not leak. However, PEG/ASB CPCM with 70% theoretical PEG content (CPCM4) in Figure 3b showed grey trace around many sample particles, and there was obvious exudation area of liquid PEG in Figure 3c after removal of the sample. It meant that there was leakage of PEG in CPCM4, and the amount of PEG in CPCM4 was excessive and exceeded the adsorption ability of ASB. Therefore, in this study, the actual contents of PEG in CPCM3 needed to be measured to verify the max weights of PEG that could be fixed in ASB.

#### 2.1.3. XRD Patterns of PEG/ASB Composites

**Figure 4:**
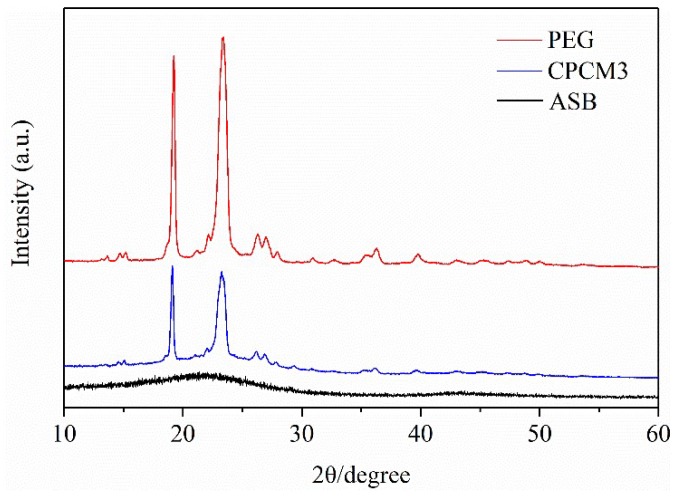
X-ray diffraction (XRD) patterns of PEG, CPCM3 and ASB. a.u.: arbitrary unit.

The X-ray diffraction (XRD) patterns of PEG, ASB and CPCM3 are displayed in Figure 4. It can be clearly seen that there were two obvious high diffraction peaks in the pattern of PEG at 19.21° and 23.39° [37], indicating that PEG showed good crystallization. For ASB, no drastically sharp diffraction peaks were observed, suggesting that ASB was non-crystalline. The pattern of CPCM3 showed the same two characteristic diffraction peaks of PEG along with the almost smooth and wide peaks of ASB, and there were no other obvious new peaks, which demonstrated that PEG was already fixed into the structure of ASB and that ASB did not influence the crystal structure of the adsorbed PEG [11]. It meant that PEG kept crystalline state in the structure of porous ASB stabilizer and the chemical compatibility between PEG and ASB was favorable. Thus, it could be speculated that chemical reactions did not occur during the CPCMs preparation between PEG and ASB [8,20,38]. However, the two peaks were lower than those of the pure PEG, suggesting that the crystallinity of CPCM3 was lower than that of the pure PEG. Compared to the pure PEG, the concentration of PEG in CPCM3 was relatively lower and ASB acted as an impurity, which caused the phenomenon of crystallization peaks decreasing and interfered with the growth of PEG crystal [2,16]. In addition, the pore structures of ASB could also limit the crystallinities of PEG in CPCMs to some extent [17].

#### 2.1.4. FT-IR Analysis of PEG/ASB Composites

**Figure 5:**
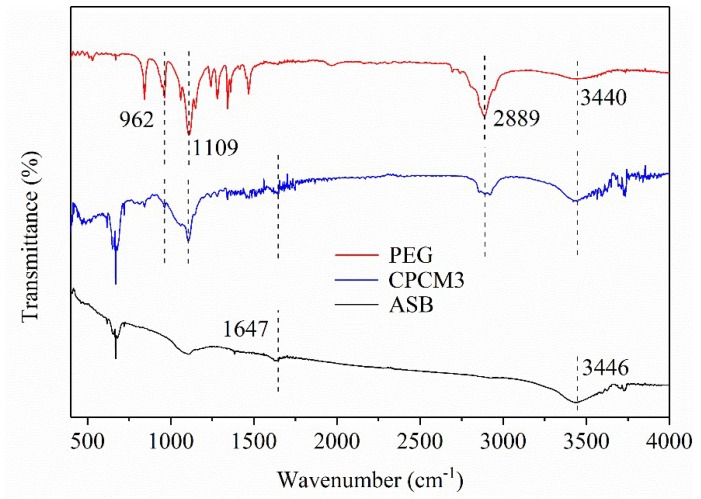
Fourier transform infrared (FT-IR) spectra of ASB, CPCM3 and PEG.

The fourier transform infrared (FT-IR) spectra of PEG, ASB and CPCM3 are displayed in Figure 5. In the biochar spectrum, two characteristic absorption peaks at 1647 cm^−1^ and 3446 cm^−1^ were observed due to the stretching vibrations of the C=C and –OH groups, respectively [31,39,40]. For PEG, stretching vibrations of the –CH_2_ functional groups were observed at 962 cm^−1^ and 2889 cm^−1^ [38]. Moreover, C–O and –OH groups could be found at 1109 cm^−1^ and 3440 cm^−1^ [41], respectively. As a result, every main peak of ASB and PEG appeared in the spectrum of CPCM3 without other new peaks, and there was no obvious shift of these main peaks as well. It was confirmed that the interaction between PEG and ASB occured through a single physical process, including the van der waal force, capillary and surface tension forces rather than chemical reactions [41,42], which was in accordance with the hypothesis suggested in the discussion of the XRD patterns.

### 2.2. Thermal Properties

#### 2.2.1. Thermal Stability of PEG/ASB Composites

**Figure 6:**
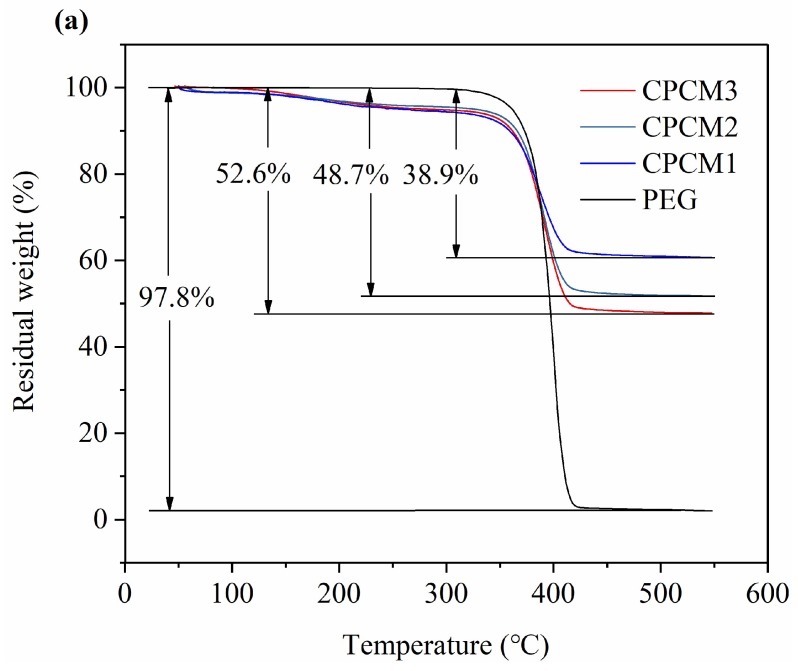

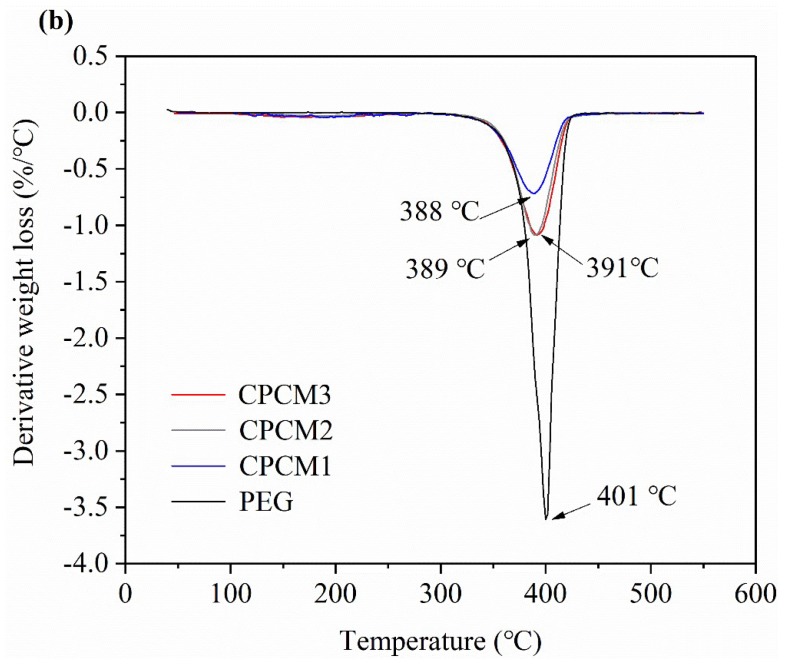
TGA curves of PEG and CPCM3 (**a**); Derivative weight loss (DTG) curves of PEG and CPCM3 (**b**).

Thermal stability is a significant property for the application of PCMs. The TGA and derivative weight loss (DTG) curves of PEG and CPCMs are displayed in Figure 6. The relevant data of TGA test are summarized in Table 1. For PEG, a slight weight loss started to appear at 282 °C. Then, a sharp weight loss occurred from approximately 354 °C to 426 °C, implying that the decomposition of PEG occurred mainly in this temperature range, and the maximum weight loss rate was observed at 401 °C in Figure 6b, suggesting that PEG molecular chains was broken. Then, the weight loss became slight again, similar to the results for temperatures lower than 282 °C, and then finally stabilized. It can be clearly seen that the trend for the TGA and DTG curves of CPCMs were similar to those of PEG curves. The differences were mainly present for the temperature points of different weight loss steps. For CPCM3, there was only a slight weight loss before 346 °C due to elimination of adsorbed water, and then the sharp weight loss started from 346 °C. Then the maximum weight loss rate was observed at 391 °C and the sharp weight loss step ended at 426 °C. In summary, no significant weight loss occurred before 310 °C in the curves of PEG and CPCMs, indicating that PEG and the PEG/ASB CPCM both had good thermal stabilities below 310 °C.

As shown in Figure 6a and Table 1, the residual weight in pure PEG was 2.2% and the weight loss was 97.8%, and the results were similar to those shown in some other reports [6,11,38,39]. For CPCM1, CPCM2 and CPCM3, the residual weights were 61.1%, 51.3% and 47.4%, respectively, and the weight losses were 38.9%, 48.7% and 52.6%, respectively. The final weight loss in CPCMs contained a slight weight loss of adsorbed water. This result also suggested that the actual contents of PEG in CPCMs were less than the theoretical proportions, which might be attributed to the insufficient adsorption of PEG by the ASB during the preparation experiment.

#### 2.2.2. Interaction between PEG and ASB

**Figure 7:**
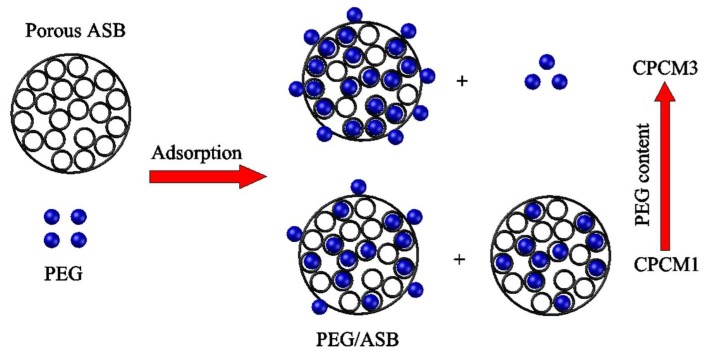
Schematic illustration of the interaction between PEG and ASB.

The schematic illustration of the interaction between PEG and ASB is displayed in Figure 7. According to the results of TGA tests in Figure 6, the PEG contents in CPCM1, CPCM2 and CPCM3 were 38.9%, 48.7% and 52.6%, respectively. For CPCM1, PEG content was low and PEG could be firmly adsorbed by ASB pore structures through capillary and surface tension forces. These forces confined the movement of PEG polymer chains and the excess ASB without adsorbing PEG increasing the restriction, and then the crystallinity of CPCM1 was limited [16]. However, when PEG content was high, as CPCM3, more PEG could be adsorbed on ASB surface compared to CPCM1 and could remain a relative free state for crystallization compared to the PEG adsorbed in ASB pores [41]. Thus, more parts of PEG/ASB CPCM could crystallize effectively with increasing PEG content, and this could bring higher enthalpy of composites. In conclusion, although ASB confined PEG movement, the liquid phase leakage problem of PEG was successfully solved thanks to ASB and ASB could also improve the shape stability of composites.

#### 2.2.3. Phase Change Behaviors of PEG/ASB Composites

**Figure 8:**
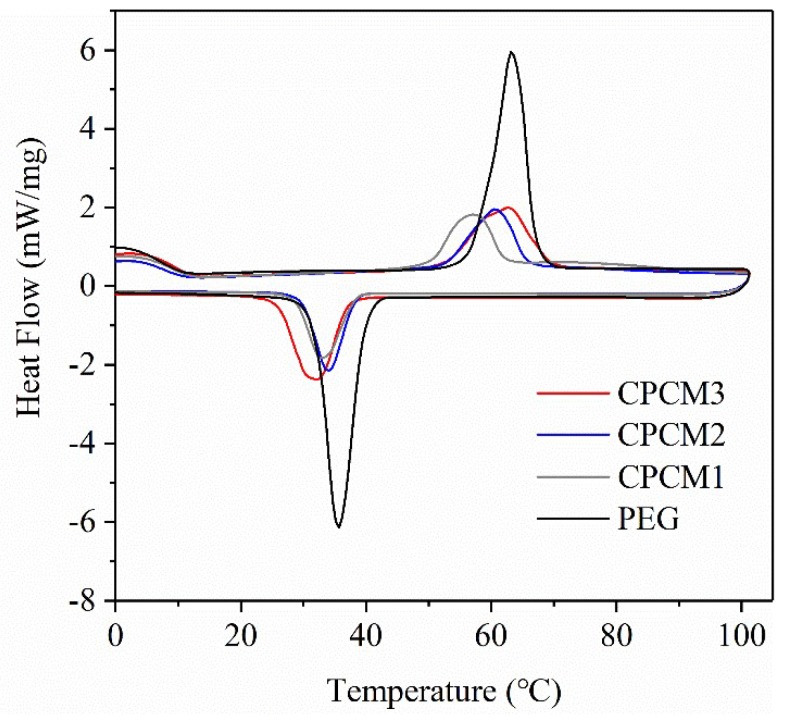
Differential scanning calorimetry (DSC) curves of PEG and CPCMs in different concentrations.

**Figure 9:**
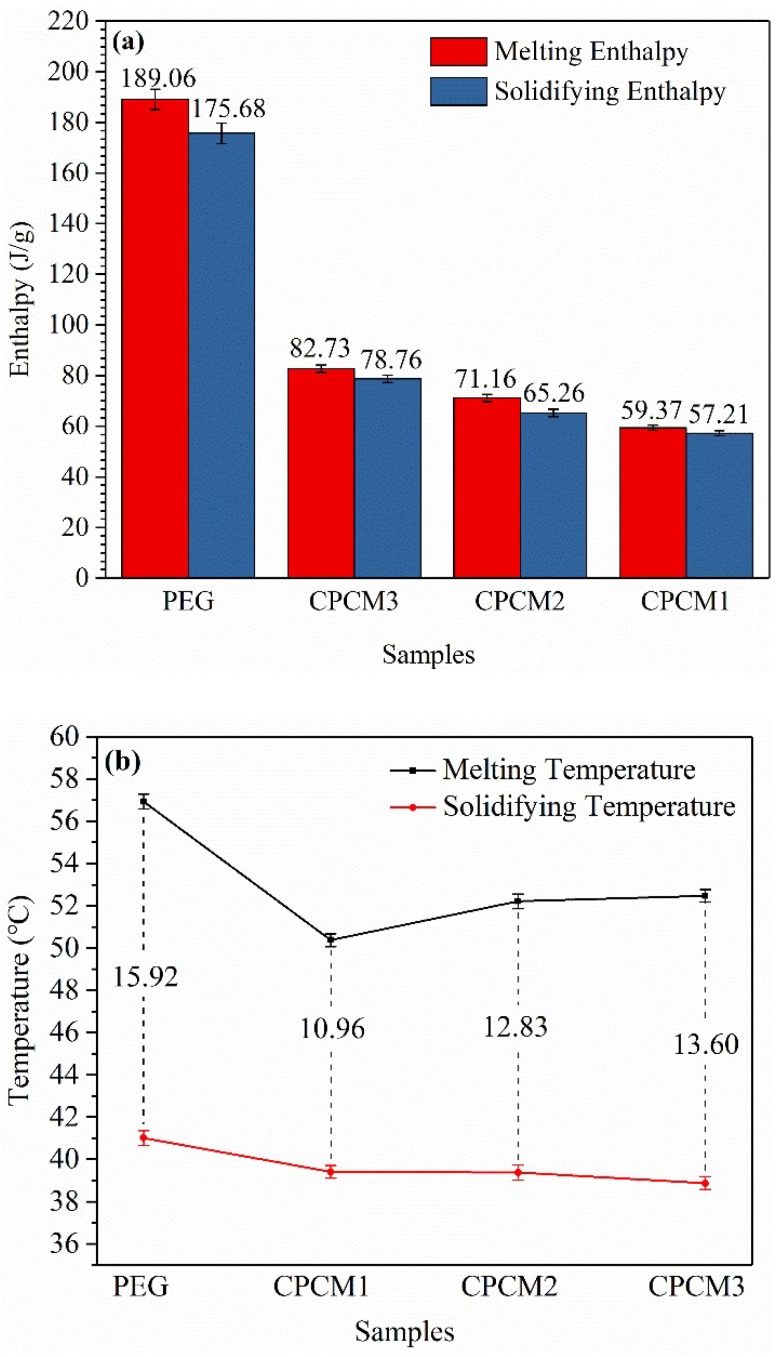
Enthalpies of melting and solidification (**a**); Supercooling of PEG and CPCMs (**b**).

The phase change behaviors of PEG and PEG/ASB CPCMs, including the phase change temperatures and latent heats, were evaluated using the DSC technique. The DSC curves of PEG and CPCMs are displayed in Figure 8, and the relevant test data are summarized in Table 2 and Figure 9a. For PEG, the melting and freezing points were 56.93 °C and 41.01 °C, respectively. The temperatures of the peaks appearing during the course of melting and freezing were 63.13 °C and 35.70 °C. It can be seen that the values of these parameters for CPCMs were lower to a different extent than those of the pure PEG, and this phenomenon may be caused by two reasons. The first reason was that the thermal conductivities of CPCMs were improved after biochar addition, and the second reason was that the PEG crystallization was interfered with due to the physical interaction, consistent with the conclusions of the XRD and FT-IR experiments [39]. The PEG contents in CPCM1, CPCM2 and CPCM3 were 38.9%, 48.7% and 52.6%, respectively. The peaks of the melting and crystallization of the CPCMs and the enthalpies of the CPCMs decreased with the decreasing PEG content, which meant that the latent heat storage capacity of the CPCMs increased with the PEG weight percentage. In addition, the latent heats of PEG were 189.06 J/g during the melting process and 175.68 J/g during the solidification process, and the corresponding values for CPCM3 were 82.73 J/g and 78.76 J/g, respectively. The enthalpies were lower than those of the ratio of PEG in the CPCM3, and this was also observed for CPCM1 and CPCM2. The physical confinement functionality of biochar on PEG may be one of the reasons for the latent heat loss discussed above, and this outcome improved the thermal stability of the composites and avoided the leakage of PEG.

The supercooling phenomenon of PEG and CPCMs can be analyzed via the test data obtained in DSC. As is shown in Figure 9b, the degree of supercooling was assessed using the difference in the values of the melting and freezing points of every sample. It can be clearly seen that the supercooling temperatures of the CPCMs were lower than that of PEG, which demonstrated that the supercooling degrees of the CPCMs could be reduced effectively after the impregnation of biochar, and this is beneficial for the practical application of CPCMs [43].

#### 2.2.4. Thermal Reliability of PEG/ASB Composites

**Figure 10:**
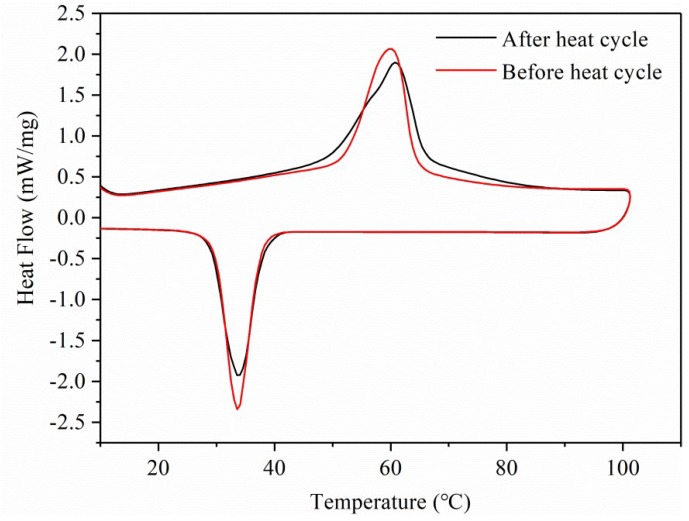
DSC curves of CPCM3 before and after the heat cycle.

In this study, heat cycle experiments test of CPCM3 were used to evaluate the thermal reliability. The heat cycle tests were conducted using an oven at 65 °C, and the cycle index was 50. The DSC curves of CPCM3 before and after the heat cycle are displayed in Figure 10. It can be seen that the two curves showed similar trend, and there were some slight differences. After the heat cycle, slight heat loss was observed for both the melting and solidification routes, which could be considered a normal phenomenon caused by the tiny leakage of PEG adsorbed on the surface of ASB. Thus, the heat cycle treatment experiments suggested that the PEG/ASB CPCMs have good thermal reliability.

#### 2.2.5. Thermal Conductivity of PEG/ASB Composites

**Figure 11:**
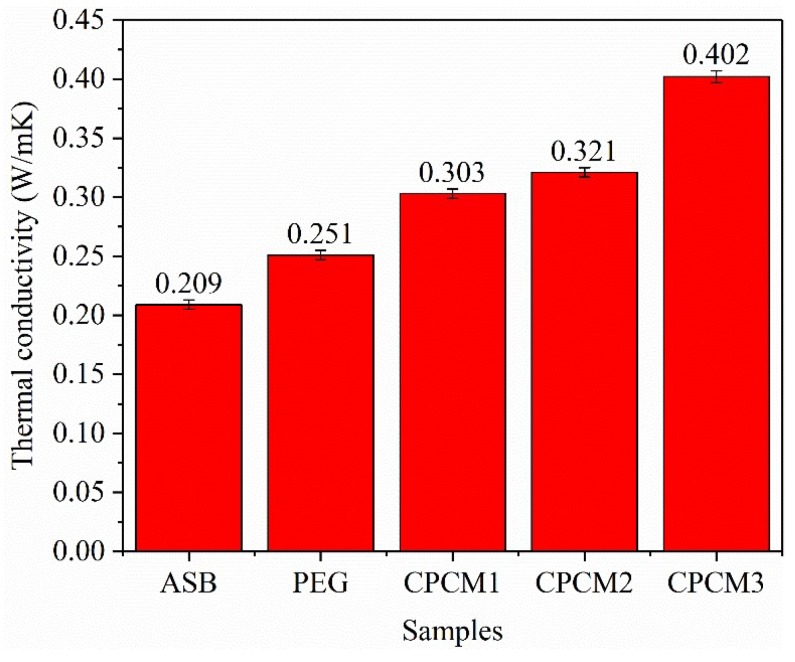
Thermal conductivity of ASB, PEG and PEG/ASB CPCMs.

The results of thermal conductivity tests of ASB, PEG and PEG/ASB CPCMs are shown in Figure 11. The values of thermal conductivity of ASB, PEG, CPCM1, CPCM2 and CPCM3 were 0.209 W/mK, 0.251 W/mK, 0.303 W/mK, 0.321 W/mK and 0.402 W/mK, respectively. It can be clearly seen that the thermal conductivities of CPCM1, CPCM2 and CPCM3 were 1.2, 1.3 and 1.6 times higher than that of the pure PEG due to the addition of ASB, and the values increased with the increase of the proportion of ASB in CPCMs, though the thermal conductivity of ASB was a bit lower than PEG. The pore structure of ASB could affect its thermal conductivity because the air was filled into the pores of ASB and then reduced its thermal conductivity, which meant that 0.209 W/mK was actually the thermal conductivity of ASB filled with air rather than that of the pure carbon. On the contrary, when ASB adsorbed PEG, ASB was beneficial for the heat conduction in PEG/ASB CPCMs and can increase the thermal conductivity of the composites, because abundant heat could transfer through the pure porous carbon skeleton and the thermal conductivity of pure ASB skeleton should be close to that of other carbon materials with high thermal conductivity, such as carbon fibers [37]. The high thermal conductivity of PCM could enhance the efficiency of energy transition and utilization during the melting and solidifying process. Therefore, the PEG/ASB CPCMs had great potential in practical thermal energy storage applications.

## 3. Materials and Methods 

### 3.1. Materials

In this study, almond shells were obtained from apricots planted in Xinjiang, China. The clean almond shells were dried at 105 °C for over 24 h and were then crushed into powder by a rotary cutting mill with high speed. Then, the particles with diameters of under 200 meshes (≤0.074 mm) were collected for biochar production. The average molecular weight of polyethylene glycol (PEG) is 4000, and it was purchased from Shanghai Aladdin Bio-Chem Technology Co LTD (Shanghai, China). Absolute ethanol was purchased from Sinopharm Chemical Reagent Co LTD (Shanghai, China).

### 3.2. Pyrolysis Procedure

**Figure 12:**
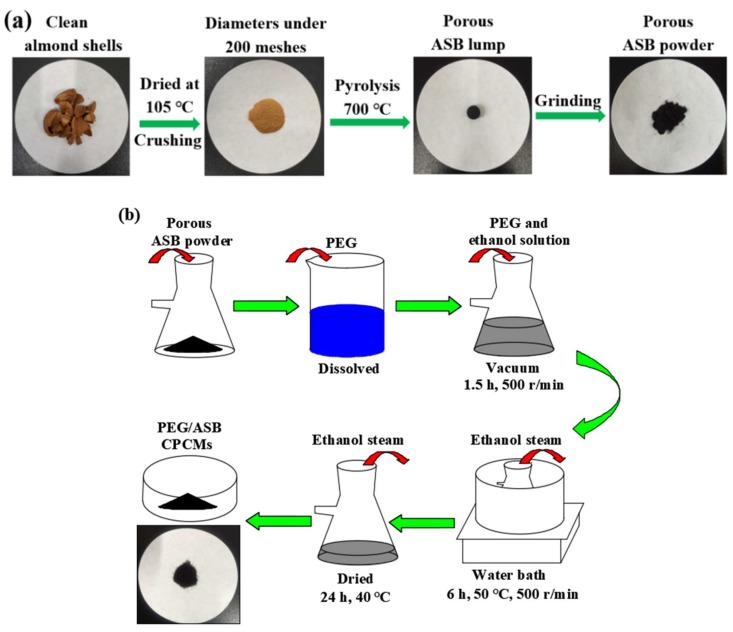
Schematic illustration of ASB preparation (**a**) and PEG/ASB CPCMs preparation (**b**).

The schematic illustration of ASB preparation is shown in Figure 12a. The pyrolysis procedure was conducted in a tube furnace under ordinary pressure, and the sustaining nitrogen (N_2_) flow was 50 mL/min. First, the heating process started at indoor temperature and was conducted at a heating rate of 10 °C/min until reaching the final pyrolysis temperature of 700 °C. Second, after this process continued for 2 h at 700 °C, the furnace was cooled down naturally to indoor temperature. Finally, the ASB lump was collected and ground into powder.

### 3.3. Preparation of PEG/ASB CPCMs

The schematic illustration of PEG/ASB CPCMs preparation is shown in Figure 12b. The simple blending and vacuum impregnation method was used for the preparation of PEG/ASB CPCMs. The proportion of PEG in the composites were designed as the theoretical calculation weights of 40%, 50%, 60% and 70%. Four PEG/ASB CPCM samples were acquired and were labeled as CPCM1, CPCM2, CPCM3 and CPCM4. First, the biochar powder was placed into a conical flask, and PEG was dissolved into absolute ethanol (approximately 0.04–0.1 g of PEG per 10 mL of absolute ethanol); then, the solution was added into the conical flask to cover the biochar powder. Second, a vacuum pump was used to get rid of the air from the composite when the solution was stirred vigorously at a rate of 500 r/min at indoor temperature, and this process was carried out for 1.5 h. Then, the conical flask was transferred into a water bath, and the solution was kept stirring at 50 °C at the same speed for 6 h; then, the composite was dried at 40 °C for over 24 h. Finally, the leakage test of composites was carried out for 10 min in an oven at 65 °C above the PEG melting temperature. As a result, it was found that when the PEG contents were 40%, 50%, 60%, there was no liquid PEG leakage, and then the actual contents of PEG in CPCMs were measured.

### 3.4. Analysis Methods

The microstructure images of ASB and PEG/ASB CPCMs were obtained by scanning electronic microscopy (SEM, FEI Company, Quanta FEG-250, Hillsboro, OR, USA). The Brunauer-Emmett-Teller (BET, Quanta, NOVA2000E, Boynton Beach, FL, USA) surface area and pore structure characteristics of ASB, including pore volume and diameter, were obtained via a nitrogen adsorption method. X-ray diffraction (XRD, HAOYUAN INSTRUMENT, DX-2700, Dandong, China) patterns of PEG, ASB and PEG/ASB CPCMs were recorded at 40 kV and 30 mA at angles ranging from 10° to 60° to evaluate their crystal behaviors. The FT-IR spectra of PEG, ASB and PEG/ASB CPCMs were acquired by a Fourier transform infrared spectrometer (FT-IR, Nicolet, Nicolet-6700, Madison, WI, USA), ranging from 400 cm^−1^ to 4000 cm^−1^. The thermal properties of PEG and PEG/ASB CPCMs, including enthalpy and phase change temperatures, were obtained by differential scanning calorimetry (DSC, NETZSCH, 200F3, Selb, Germany). The samples were first heated from 0 °C to 100 °C and were then cooled from 100 °C to 0 °C at the rate of 10 °C/min rate under continuous nitrogen flow. The thermal stabilities of PEG and PEG/ASB CPCMs were analyzed by thermogravimetric analysis (TGA, NETZSCH, 209F3, Selb, Germany) from indoor temperature to 550 °C under a nitrogen atmosphere and with a heating rate of 10 °C /min. The thermal conductivity of PEG, ASB and PEG/ASB CPCMs were measured at 35 °C by a Xenon lamp flash thermal conductivity meter (TA Instruments, DXF-500, New Castle, DE, USA).

## 4. Conclusions

In summary, PEG/ASB form-stable CPCM was developed via the simple blending and vacuum impregnation method. The environmental and low cost ASB was produced from almond shells via a simple slow pyrolysis and it firstly acted as the supporting material of PEG. To study the characteristics of ASB, PEG and PEG/ASB, the structural properties were investigated by XRD, FT-IR, SEM and BET, and the thermal properties were examined using DSC, TGA and thermal conductivity test. The BET results indicated that ASB could be used as a good adsorbent material. PEG was fixed into ASB successfully, and there was no chemical reaction between PEG and ASB but rather physical interactions only. The latent heat storage capacities of the PEG/ASB were shown to increase as the PEG contents increased, and the prepared PEG/ASB possessed good thermal stabilities below 310 °C. The addition of ASB greatly improved the thermal conductivity of PEG/ASB compared to the pure PEG. In conclusion, the PEG/ASB could be used as a reliable energy storage material due to its good latent heat storage capacity and shape stability.

## Figures and Tables

**Table 1:** TGA test results of PEG and CPCM3.

Samples	Onset (°C)	Peak (°C)	End (°C)	Residual Weight (%)
PEG	354	401	426	2.2
CPCM3	346	391	426	47.4
CPCM2	345	389	424	51.3
CPCM1	340	388	420	61.1

**Table 2:** Phase change characteristics of CPCMs and PEG.

Samples	PEG Contents ^a^	Melting			Solidification		
		*T_mp_*^b^ (°C)	*T_m_*^c^ (°C)	*H_m_*^d^ (J/g)	*T_sp_*^e^ (°C)	*T_s_*^f^ (°C)	*H_s_*^g^ (J/g)
PEG	100%	63.13	56.93	189.06	35.70	41.01	175.68
CPCM3	60%	62.76	52.47	82.73	31.97	38.87	78.76
CPCM2	50%	60.49	52.21	71.16	34.06	39.38	65.26
CPCM1	40%	57.04	50.37	59.37	33.03	39.41	57.21

^a^ theoretical PEG contents; ^b^ ***T_mp_***: melting peak temperature; ^c^ ***T_m_***: melting temperature; ^d^ ***H_m_***: melting enthalpy; ^e^ ***T_sp_***: solidifying peak temperature; ^f^ ***T_s_***: solidifying temperature; ^g^ ***H_s_***: solidifying enthalpy.
